# Stability of the Subaxial Spine after Penetrating Trauma: Do Classification Systems Apply?

**DOI:** 10.1155/2018/6085962

**Published:** 2018-10-09

**Authors:** Jackson Rucker Staggers, Thomas Elliot Niemeier, William E. Neway, Steven Michael Theiss

**Affiliations:** Division of Orthopaedic Surgery, University of Alabama at Birmingham, Birmingham, Alabama, 35205, USA

## Abstract

**Objective:**

Blunt spinal trauma classification systems are well established and provide reliable treatment algorithms. To date, stability of the spine after civilian gunshot wounds (CGSWS) is poorly understood. Herein, we investigate the validity of trauma classification systems including the Thoracolumbar Injury Classification and Severity Score (TLICS), Subaxial Cervical Spine Injury Classification and Severity Score (SLIC), and Denis' three-column model when applied to spinal penetrating trauma from gunshots, while secondarily evaluating stability of these injuries.

**Methods:**

Gunshot injuries to the spine were identified from an institutional database from ICD-nine codes. Trauma scorings systems were applied using traditional criteria. Neurologic compromise and spinal stability were evaluated using follow-up clinic notes and radiographs.

**Results:**

Thirty-one patients with CSGSW were evaluated. There was an equal distribution of injuries amongst the spinal levels and spinal columns. Twenty patients had neurological deficits at presentation. Eight patient had a TLICS score >4. Three patients had a SLIC score >4. One patient had surgical treatment. Nonoperative treatment did not lead to spinal instability or adverse outcomes in any cases. The posterior column had a high correlation with neurologic compromise, though not statistically significant (p=.118).

**Conclusions:**

The TLICS, SLIC, and three-column classification systems cannot be applied to CSGSW to quantify injury severity, predict outcomes, or guide treatment decision-making. Despite significant neurologic injuries and disruption of multiple spinal columns, CSGSW do not appear to result in unstable injuries requiring operative intervention. Further research is needed to identify the rare spinal gunshot injury that would benefit from immediate surgical intervention.

## 1. Introduction

Civilian spinal gunshot wounds (CSGSW) are an increasingly common injury and carry significant morbidity and mortality [1–4]. Annually, CSGWS are the third most common cause of spinal injury and account for approximately 13-17% of all traumatic spinal injuries [1, 2, 5–9]. Despite the severity and frequency of CSGSW, there is little agreement on the universal classification and management of these injuries since surgeons continue to treat patients based on institutional, geographical, and surgeon preference [10–12]. As a result, numerous classification systems and stability concepts have been applied to these injuries in an attempt to improve care and optimize outcomes.

Three of the more popular spinal classification systems are the Thoracolumbar Injury Classification and Severity Score (TLICS), Subaxial Cervical Spine Injury Classification and Severity Score (SLIC), and Denis' three-column model. TLICS was introduced in 2005 and is a point-based system that utilizes the morphology of the injury, integrity of the posterior ligamentous complex (PLC) and neurologic status to evaluate an injury [10]. SLIC is another point-based system that was introduced in 2007 and is based on injury morphology, integrity of the disco-ligamentous complex, and neurologic status [13–15]. Both TLICS and SLIC give an injury score that correlates with injury severity, allowing the provider to quantify injury severity and guide treatment [13–15]. Denis' three-column model was introduced in 1983 and is based on radiographic findings [16, 17]. It divides the spine into three anatomic columns and defines instability as disruption of 2 or more columns [16, 17]. Although the three-column system provides an intuitive nomenclature for describing spinal injuries, it does not provide prognostic information or guide clinical decision-making [10].

These systems have been validated in many studies as reliable and reproducible for blunt force trauma; however, to date their use in penetrating trauma has not been validated [4, 10, 13–15]. Currently there are no spinal classification systems that were designed for penetrating trauma, and data in the literature is limited. This study seeks to assess the utility and legitimacy of the TLICS, SLIC, and three-column classification systems to quantify injury severity, predict outcomes, and guide treatment decision-making for CSGWS, while secondarily evaluating stability of these injuries.

## 2. Materials and Methods

After institutional review board (IRB) approval was obtained, we conducted a retrospective cohort review of patients who sustained low-velocity gunshot injuries to the spine from 2003-2016 using ICD-nine codes. Patients were treated by orthopedic and neurologic surgeons from a single level-one trauma center. All treatments were non-randomized and were at the discretions of the treating surgeon. Inclusion criteria included gunshot injuries that involved any aspect of the cervical, thoracic, lumbar, or sacral spine. Single and multiple gunshot injuries were included. Patients with non-spinal gunshot injuries were included as long as the injuries did not affect management of their spinal injuries. Patients who were unable to provide neurological exam due to clinical condition or with less than one month of follow up were excluded from the study. Also, patients without computed tomography (CT) imaging on the date of injury or radiographic or CT imaging at final follow up were excluded.

Standard patient demographic information was reviewed, including: age, date of injury, fracture morphology, fracture level, treatment, neurologic status, and final follow up data. Neurologic status was assessed using the American Spine Injury Association (ASIA) classification [18]. In some instances, a single ballistic injury resulted in multiple spinal level injuries due to an oblique sagittal tract. For these patients and for those with multiple spinal gunshot injuries, all spinal levels and columns involved were counted, but only the most severe injury was used for neurological and classification analysis. Initial CT evaluation was performed to assess fracture morphology, injury level, and coronal and sagittal alignment. The three-column system of spine stability was applied as classically described by Denis [16]. The Subaxial Cervical Spine Injury Classification and Severity Score (SLIC) and Thoracolumbar Injury Classification and Severity Score (TLICS) were calculated based off original papers by Vaccaro [10, 13]. In order to classify complex fracture patterns according to the pre-defined morphologic patterns of SLIC and TLICS systems, fractures that only affected the anterior column of the vertebral body were described as compression fractures and were assigned one point. Fractures that involved the anterior and middle columns were described as burst fractures and assigned two points. Injuries that involved the vertebral body and the posterior elements were assigned four points. Isolated posterior column injuries were given a morphologic score of zero. Patients with intact posterior elements were given no points whereas injuries that caused complete destruction of the posterior elements were classified as having a suspected PLC injury and given two points. On final follow-up, radiographs were evaluated for sagittal, coronal, and axial spinal alignment and final neurologic status was recorded.

Demographic information was evaluated using descriptive statistical analysis. Further statistical analysis was done using chi-squared tests. A* p *<0.05 was considered statistically significant.

## 3. Results

Thirty-one patients (27 males, 4 females) with 33 spinal gunshot injuries met the inclusion criteria for the study. Mean age was 34.0+/- 14.8 years (range 17-63 years) with an average follow up of 2.0 +/- 2.6 years (range 1-99 months). Demographic, fracture, and neurologic information is shown in Table 1. ASIA neurological status was recorded in all patients with 20 of the 31 patients (64.5%) having neurologic deficits (ASIA A-D) and 11 patients without any neurologic deficits (ASIA E) immediately after presentation. Complete spinal cord injury (ASIA A) was the most commonly encountered deficit (9 patients). There was a nearly equal representation of CSGSW among cervical, thoracic, and lumbar regions, but only one injury that involved the sacral spine. Twenty-one patients (67.7%) had injuries involving multiple vertebrae. Two patients (6.5%) had isolated intervertebral disk involvement. All but one patient were treated nonoperatively.

On follow up, no patients displayed significant change in spinal alignment or neurologic status. One patient with concomitant bowel injury developed spondylodiscitis and a progressive lumbar kyphosis secondary to near complete vertebral body collapse. This patient's CGSW injury was isolated to the posterior elements and their deformity was deemed unrelated to instability secondary to the initial trauma.

### 3.1. Thoracolumbar Injury Classification and Severity Score (TLICS)

TLICS scores for each patient with thoracolumbar involvement are summarized in Table 2. Eleven patients of the 23 patients with thoracolumbar injuries (47.8%) had a TLICS score of 0-3 suggesting conservative therapy. Four patients (17.4%) scored a 4 suggesting that surgery is up to the surgeon's discretion. Eight patients (34.8%) had a TLICS score of greater than 4 suggesting surgical stabilization is appropriate. Despite the high number of patients for whom surgery is indicated according to TLICS, none had surgery.

### 3.2. The Subaxial Cervical Spine Injury Classification and Severity Score (SLIC)

SLIC scores for each patient with cervical spine involvement are summarized in Table 3. Five of the 8 patients with cervical injuries (62.5%) had a SLIC score of 0-3, suggesting conservative therapy. Three patients (37.5%) scored greater than 4, suggesting surgical stabilization is appropriate. Only 1 patient (12.5%) with a SLIC score of 5 had surgical stabilization.

### 3.3. Denis' Three-Column Model

**Figure 1:**
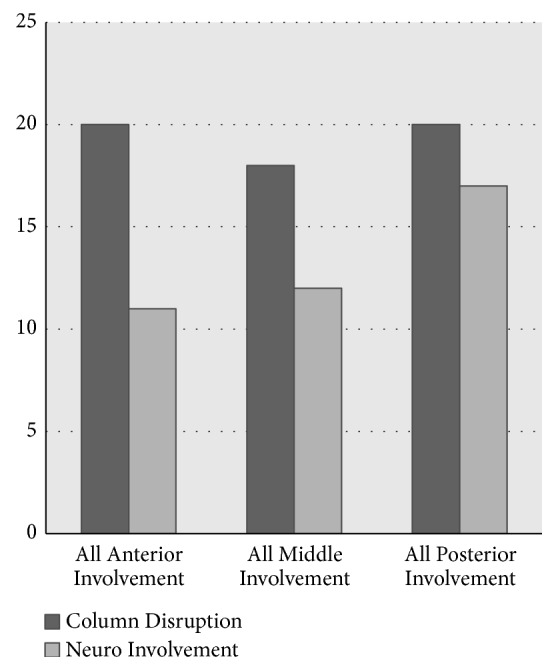
Correlation of neurologic injury and column disruption in Denis' three column stability system.

**Figure 2:**
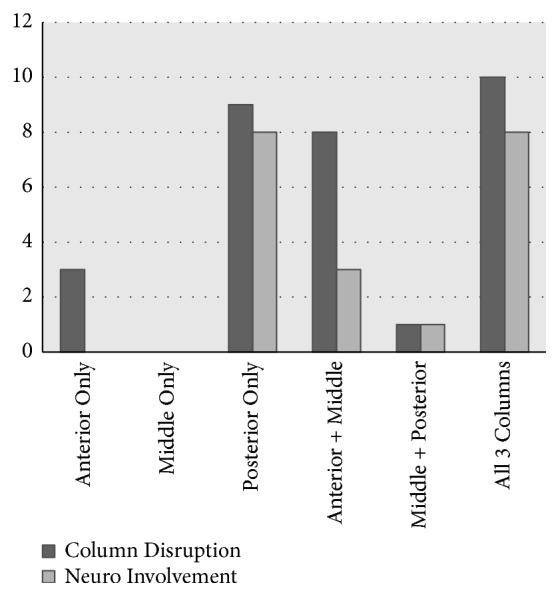
Correlation of neurologic injury and column disruption in Denis' three column stability system.

Table 4 summarizes the number of injuries per column as it pertains to Denis' three-column model. There was a near equal distribution of one-column (12), two-column (9), and three-column (10) injuries, as well as a near equal involvement of anterior (20), middle (18), and posterior columns (20). The association between column involvement and neurologic injury is described in Figures 1 and 2. One and two-column injuries involving the anterior and middle columns had a low correlation with neurological involvement. Though it was not statistically significant, there was a high correlation of the posterior column with neurologic involvement (*p* =0.118). Of note, three-column injuries also had a high correlation with neurological involvement.

## 4. Discussion

Contemporary understanding of spinal injuries and their optimal management is continuously evolving. Classification systems have been used for decades for spinal trauma as a helpful resource, but there is little agreement on the universal classification across a broad spectrum of injuries. The TLICS, SLIC, and the three-column classifications have been validated in blunt trauma and provide useful information on the mechanical stability of the spine [4, 10, 13–15]. The current study investigated the utility of trauma classification systems in penetrating gunshot injuries, a topic that has yet to be discussed.

Ideally, a classification system should be able to provide prognostic information and help guide treatment. Application of TLICS and SLICS scores system were not able to predict injury severity or instability in our study. Eight patients (34.8%) with thoracolumbar injuries and 3 patients (37.5%) with cervical injuries had a TLICS or SLIC severity score that equated with recommendation for surgery. All but one of these patients were successfully managed conservatively with the one exception undergoing early surgical stabilization.

**Figure 3:**
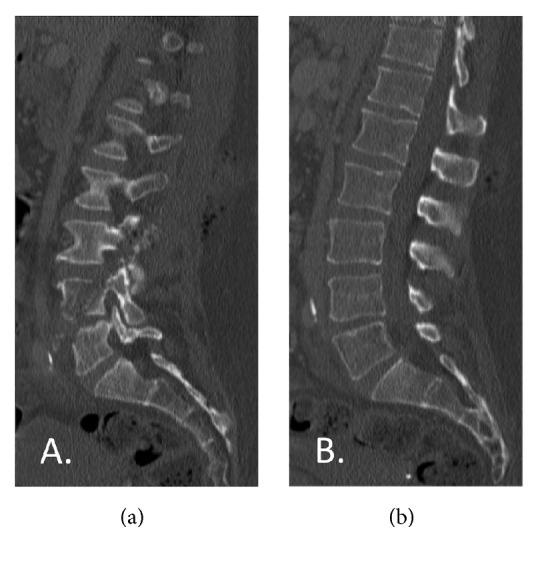
(a) Parasagittal image demonstrating bullet tract involving multiple vertebral bodies and articulating facets joints. (b) Midsagittal image on the same patient seen in image 2A showing intact alignment despite CSGSW.

Fracture morphology was a major limiting factor for the general utility of TLICS and SLIC systems since penetrating wounds do not usually result in classic fracture patterns. By definition, burst and compression fractures result in a loss of vertebral body height. In our cohort, nearly all of the CSGSW retained normal vertebral body alignment, intuitively making them more stable than their nomenclature suggested (Figure 3). This increased stability is thought to be the result of nearby supporting structures that are more likely to be maintained in penetrating trauma.

**Figure 4:**
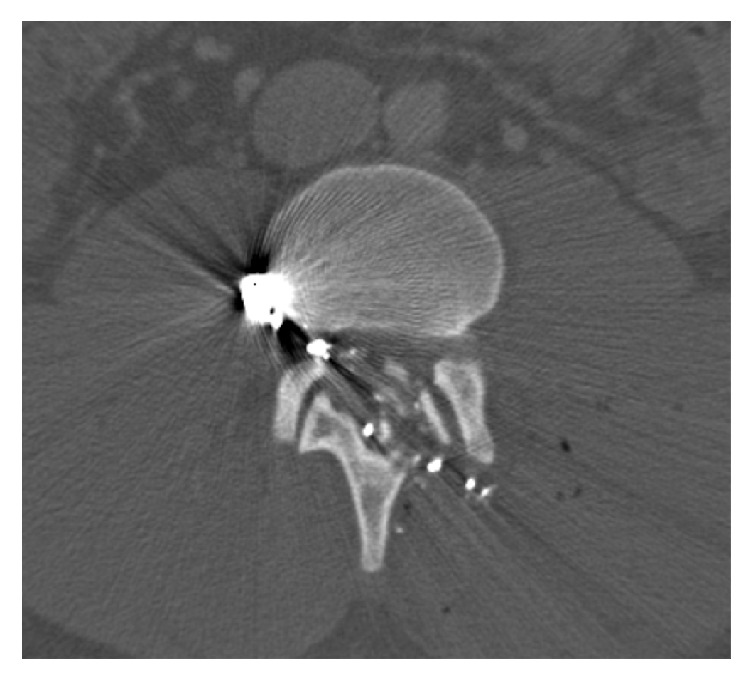
Bullet seen traversing spinal canal in a patient with complete neurologic injury (ASIA A).

In TLICS and SLIC, neurologic status is used as a critical indicator of stability, but this may not have the same utility in CSGSW as it does in blunt force trauma. In our study, roughly two-thirds of patients had neurologic involvement without any patients showing spinal instability. This data is consistent with previous work by Bumpass et al. who showed a high rate of neurologic compromise despite low rates of biomechanical instability with CSGSW [19]. Prior studies suggest that in CSGSW, neural and spinal cord damage are generally due to direct impact, thermal energy, or blast effect (Figure 4) rather than compression or tension as seen in blunt force trauma [1, 20].

The Denis three-column spinal stability model provided no insight into instability or prognostic information for CSGSWS. Ten patients (32.2%) sustained three-column injuries as classified by Denis but all were successfully treated without surgical treatment. Though the posterior column had a high correlation with neurologic damage, this is believed to be related to classification system's basis on anatomy and the location of the spinal cord.

Our data is consistent with past studies supporting the inherit stability of the spine after CSGSW [2, 19, 21–23]. The authors of the current study find no value in classifying penetrating injuries with conventional spinal classification systems as they provide no appreciated information on mechanical stability. As previous studies have shown surgery frequently correlates with an increase in complications without improvement in outcome [19], the role of surgery should be limited to the rare patient with overt mechanical instability.

Our study is not without limitation, including its retrospective study design with a limited number of patients. Additionally, by design our study evaluates three spine trauma classification systems that were specifically designed for blunt force trauma in the setting of penetrating trauma. Though this provides little novel information, our study closes the literature gap on this topic and confirms current practices relating to penetrating trauma of the spine. A small number of patients were lost to follow-up and neurological exam required data extraction from numerous outpatient charts that were at times incomplete. Additionally, bullet fragments on CT scan serve a potential source of error as they proved to be a challenge in assess fracture morphology secondary to artifact.

## 5. Conclusion

We conclude that the TLICS, SLIC, and three-column classification systems cannot be applied to CSGSW to quantify injury severity, predict outcomes, or guide treatment decision-making. Despite significant neurologic injuries and disruption of multiple spinal columns, CSGSW do not appear to result in unstable injuries requiring immediate surgical stabilization. TLICS and SLIC grossly over-indicate surgery for many patients that actually did well when treated conservatively. Our data suggests that refraining from operative treatment does not result in worse outcomes; therefore, we propose that CSGSW warrant a trial of nonoperative management after injury. Further research is suggested to find the rare injury that would benefit from immediate surgical stabilization or debridement.

## Figures and Tables

**Table 1:** Demographic and injury data.

	**Mean**	**SD**
**Age (yrs) **	34.0	14.8

	**Number**	%** of Patients**

**Sex**		

Male	27	87.1

Female	4	12.9

**Spinal Levels Involved**		

Cervical	8	25.8

Thoracic	14	45.2

Lumbar	13	41.9

Sacral	1	3.2

**Neurologic Grade**		

ASIA A	9	28.0

ASIA B	2	6.0

ASIA C	3	9.0

ASIA D	6	19

ASIA E	11	34.0

**Treatment**		

Surgery	1	3.2

Nonoperative	30	96.8

**Stability**		

Stable	31	100.0

Unstable	0	0.0

**Table 2:** TLICS Score.

	**Thoracic Spine**	**Lumbar Spine**	**Total**
TLICS Score 0-3	4	7	11

TLICS Score 4	3	1	4

TLICS Score >4	4	4	8

**Table 3:** SLIC Scores.

	**Cervical Spine**
SLIC Score 0-3	5

SLIC Score 4	0

SLIC Score >4	3

**Table 4:** Denis Classification.

	**Number**	%
**Number of Columns Involved**		

1 Column	12	38.7

2 Columns	9	29.0

3 Columns	10	32.2

**Column Injuries**		

Anterior	20	52.6

Middle	18	47.4

Posterior	20	52.6

**Columns Involved**		

Anterior only	3	7.9

Middle only	0	0.0

Posterior only	9	23.7

Anterior/Middle	11	28.9

Posterior/Middle	1	2.6

Anterior/Middle/Posterior	10	26.3

## Data Availability

All results and conclusions of the current study can be explained through the tables published and referenced in the manuscript. The authors decline publishing raw data in order to protect patient information.
